# Prepandemic Prevalence of Dietary Supplement Use for Immune Benefits

**DOI:** 10.1001/jamanetworkopen.2024.59291

**Published:** 2025-02-11

**Authors:** Margaret A. Nagai-Singer, Edwina A. Wambogo, Stefan M. Pasiakos, Jaime J. Gahche

**Affiliations:** 1 Office of Dietary Supplements, National Institutes of Health, Bethesda, Maryland

## Abstract

This cross-sectional study of a national survey of US adults examines the prevalence of use of dietary supplements for perceived immune system benefits.

## Introduction

Dietary supplement use is common in the US,^1^ and over 25 000 supplement labels in the Dietary Supplement Label Database (DSLD) display the word *immune* or *immunity*, suggesting immune benefits are a prevalent and marketable motivation for dietary supplement use.^2^ These motivations may impact nutrient intake and modify health behaviors. However, Crawford et al^3^ found dietary supplement labeling related to immune benefits can be misleading. Utilizing the most recent National Health and Nutrition Examination Survey (NHANES) data, this study investigates the prevalence of using dietary supplements to prevent colds or boost the immune system (herein referred to as perceived immune benefits), and if label claims or health care professional recommendations influence use of dietary supplements for perceived immune benefits.

## Methods

In this cross-sectional study, the DSLD and nationally representative data from the January 2017 to March 2020 prepandemic NHANES were used to identify the prevalence and characteristics of those reporting use of dietary supplements for perceived immune benefits, label claims related to immune benefits,^2^ and use due to health care professional recommendation. Pairwise differences were compared using *t* tests and linear trends using orthogonal polynomials (2-sided *P* < .05). Statistical analyses were performed with SAS survey procedures in SAS version 9.4 (SAS Institute Inc) and SUDAAN version 11.0 (RTI International).

NHANES was approved by the National Center for Health Statistics ethics review board and written consent was obtained from adults and from parents of participants under the age of 18 years. This study followed the Strengthening the Reporting of Observational Studies in Epidemiology (STROBE) reporting guideline. Additional details can be found in Supplement 1.

## Results

A total of 15 560 persons were included in analysis. From January 2017 to March 2020, 11.0% (95% CI, 9.8%-12.2%) of the US population used a dietary supplement for perceived immune benefits. Prevalence increased as education, family income, food security, self-rated diet quality, and self-rated overall health improved. Prevalence also differed by age and was higher among those who identified as non-Hispanic Black, non-Hispanic White, and other (Table).

**Table.** Prevalence of Dietary Supplement Use for Perceived Immune Benefits^a^

Characteristic	Total use for perceived immune benefits			Use exclusively for perceived immune benefits^b^			Includes label claims related to immune benefits^b^			Use due to doctor recommendation^b^		
	Sample size	% (95% CI)	*P* value for linear trend	Sample size	% (95% CI)	*P* value for linear trend	Sample size	% (95% CI)	*P* value for linear trend	Sample size	% (95% CI)	*P* value for linear trend
Total	15 560	11.0 (9.8-12.2)	NA	1487	28.3 (24.4-32.5)	NA	886	60.1 (54.0-65.9)	NA	1483	16.6 (13.7-19.8)	NA
Sex												
Male	7721	10.4 (9.1-11.9)	NA^c^	684	26.1 (20.9-32.2)	NA^c^	414	63.3 (54.9-71.0)	NA^c^	681	15.8 (12.4-19.9)	NA^c^
Female	7839	11.5 (10.1-13.1)		803	30.2 (25.0-35.8)		472	57.3 (49.4-64.8)		802	17.2 (13.1-22.4)	
Age range, y												
0-11	4588	9.7 (8.6-10.8)^d^	<.001	389	16.9 (13.3-21.2)^e^	<.001	311	59.0 (50.5-66.9)	.17	389	11.3 (7.8-15.9)^f^	<.001
12-19	1862	6.8 (5.6-8.4)^g^		107	17.7 (11.2-26.6)^h^		61	71.5 (59.4-81.2)		107	16.1 (6.5-34.5)^i^	
20-59	5920	10.9 (9.4-12.7)^j^		600	27.4 (22.8-32.6)^k^		341	61.4 (53.1-69.0)		599	12.6 (9.6-16.3)^l^	
≥60	3190	14.1 (11.9-16.7)^m^		391	38.7 (30.1-48.1)^n^		173	53.9 (42.7-64.7)		388	27.4 (20.6-35.5)^o^	
Race and Hispanic origin												
Hispanic	3534	8.1 (6.9-9.5)^p^	NA^c^	253	27.0 (21.0-33.9)	NA^c^	137	53.1 (43.3-62.6)	NA^c^	252	16.4 (11.5-22.8)	NA^c^
Non-Hispanic Asian	1638	6.7 (5.5-8.2)^q^		107	37.5 (27.4-48.7)^r^		45	65.8 (46.7-80.9)^i^		106	11.9 (4.6-27.6)^i^	
Non-Hispanic Black	4098	10.2 (8.8-11.8)^s^		399	18.2 (13.2-24.7)^t^		257	63.6 (55.6-70.8)		398	15.1 (11.4-19.6)	
Non-Hispanic White	5271	12.1 (10.4-14.1)^u^		593	30.7 (25.3-36.8)^v^		353	60.7 (52.5-68.3)		592	17.5 (13.6-22.4)	
Other^w^	1019	14.7 (11.0-19.3)^x^		135	17.9 (7.6-36.7)^i^		94	58.7 (39.1-75.9)^i^		135	11.9 (5.3-24.8)^i^	
Education (ages ≥20 y)												
High school or below	3985	8.1 (6.2-10.6)^y^	NA^c^	287	34.5 (26.2-43.9)	NA^c^	132	58.8 (47.9-69.0)	NA^c^	286	16.5 (11.4-23.1)	NA^c^
Some college or college graduate	5232	14.1 (12.3-16.0)^g^		712	29.7 (25.0-34.8)		387	59.6 (51.6-67.2)		709	17.7 (13.5-22.8)	
Family income												
<130% FPL	4516	7.2 (5.6-9.1)	<.001^z^	286	21.5 (14.0-31.6)	.03^z^	175	53.6 (45.3-61.6)	.20^z^	285	15.1 (10.7-20.8)	.66^z^
130%-350% FPL	5027	11.0 (9.3-12.9)		535	24.2 (19.4-29.9)		314	58.0 (50.3-65.4)		532	15.2 (11.0-20.5)	
>350% FPL	3816	13.5 (11.5-15.8)		494	33.9 (27.2-41.3)		305	62.7 (52.4-71.9)		494	16.9 (11.1-24.8)	
Household food security												
Very low or low	3611	9.0 (7.0-11.4)	.02^z^	272	21.9 (15.8-29.6)	.12^z^	169	54.4 (47.0-61.6)	.20^z^	272	10.1 (6.4-15.6)	.06^z^
Marginal	2197	8.2 (6.7-10.0)		183	31.0 (22.9-40.6)		110	60.1 (48.0-71.1)		182	12.4 (7.8-19.1)	
Full	8668	12.0 (10.4-13.7)		930	29.6 (24.4-35.5)		556	60.5 (53.0-67.5)		927	17.5 (13.2-22.9)	
Diet quality (ages ≥16 y)												
Poor or fair	3349	8.9 (7.3-10.7)	<.001^z^	281	33.0 (24.3-43.2)	.93^z^	134	66.9 (51.2-79.6)^i^	.39^z^	281	16.5 (11.4-23.3)	.86^z^
Good	3966	11.2 (9.1-13.6)		395	27.2 (20.4-35.2)		216	58.7 (46.2-70.2)		393	17.6 (12.9-23.6)	
Very good or excellent	2875	14.5 (12.8-16.3)		372	32.5 (25.5-40.3)		193	57.8 (45.8-68.9)		370	17.3 (12.1-24.1)	
Overall health (ages ≥16 y)												
Poor or fair	2603	8.7 (7.1-10.7)	.002^z^	210	39.3 (30.6-48.7)	.02^z^	95	50.5 (36.2-64.7)^i^	.24^z^	208	28.3 (20.6-37.5)	.007^z^
Good	4665	10.7 (8.9-12.7)		434	26.7 (21.0-33.2)		248	59.4 (48.8-69.2)		434	20.1 (14.9-26.7)	
Very good or excellent	8282	11.8 (10.5-13.2)		842	26.8 (21.2-33.2)		542	61.6 (54.0-68.7)		840	12.2 (8.3-17.6)	

Abbreviations: FPL, federal poverty level; NA, not applicable.

^a^ Sample size is unweighted and represents the total number of respondents per group. Percentages are weighted and represent the proportion of the group using dietary supplement for the purpose outlined in the column headings.

^b^ Of those taking dietary supplements for perceived immune benefits.

^c^ Pairwise tests only (statistical significance set at *P* < .05).

^d^ Statistically different from ages 12-19 years (*P* < .001) and ≥60 years (*P* = .003).

^e^ Statistically different from ages 20-59 years (*P* = .002) and ≥60 years (*P* < .001).

^f^ Statistically different from ages ≥60 years (*P* < .001).

^g^ Statistically different from ages 0-11 years (*P* < .001), 20-59 years (*P* = .001), and ≥60 years (*P* < .001).

^h^ Statistically different from ages 20-59 years (*P* = .04) and ≥60 years (*P* < .001).

^i^ Statistically unreliable.

^j^ Statistically different from ages 12-19 years (*P* = .001) and ≥60 years (*P* = .008).

^k^ Statistically different from ages 0-11 years (*P* = .002), 12-19 years (*P* = .04), and ≥60 years (*P* = .03).

^l^ Statistically different from ages ≥60 years (*P* = .002).

^m^ Statistically different from ages 0-11 years (*P* = .003), 12-19 years (*P* < .001), and 20-59 years (*P* = .008).

^n^ Statistically different from ages 0-11 years (*P* < .001), 12-19 years (*P* < .001), and 20-59 years (*P* = .03).

^o^ Statistically different from ages 0-11 years (*P* < .001) and 20-59 years (*P* = .002).

^p^ Statistically different from non-Hispanic White (*P* = .003), non-Hispanic Black (*P* = .03), and other (*P* = .007).

^q^ Statistically different from non-Hispanic White (*P* < .001), non-Hispanic Black (*P* = .002), and other (*P* < .001).

^r^ Statistically different from non-Hispanic Black (*P* = .003).

^s^ Statistically different from non-Hispanic Asian (*P* = .002), Hispanic (*P* = .03), and other (*P* = .04).

^t^ Statistically different from non-Hispanic White (*P* = .004) and non-Hispanic Asian (*P* = .003).

^u^ Statistically different from non-Hispanic Asian (*P* < .001) and Hispanic (*P* = .003).

^v^ Statistically different from non-Hispanic Black (*P* = .004).

^w^ Other includes persons who identified as more than 1 race or ethnicity.

^x^ Statistically different from non-Hispanic Black (*P* = .04), non-Hispanic Asian (*P* < .001), and Hispanic (*P* = .007).

^y^ Statistically different from some college or college degree (*P* < .001).

^z^ Linear trends only (statistical significance set at *P* < .05).

Among those taking dietary supplements for perceived immune benefits, 28.3% (95% CI 24.4%-32.5%) reported using a dietary supplement exclusively for perceived immune benefits. Prevalence of using a dietary supplement exclusively for perceived immune benefits increased as age and family income increased and as self-rated overall health declined, and it differed by race and Hispanic origin (Table). The majority of those taking dietary supplements for perceived immune benefits reported at least 1 additional reason for use, of which the most common was to maintain health or stay healthy (40.0%; 95% CI, 36.5%-43.7%) (Figure).

**Figure.**
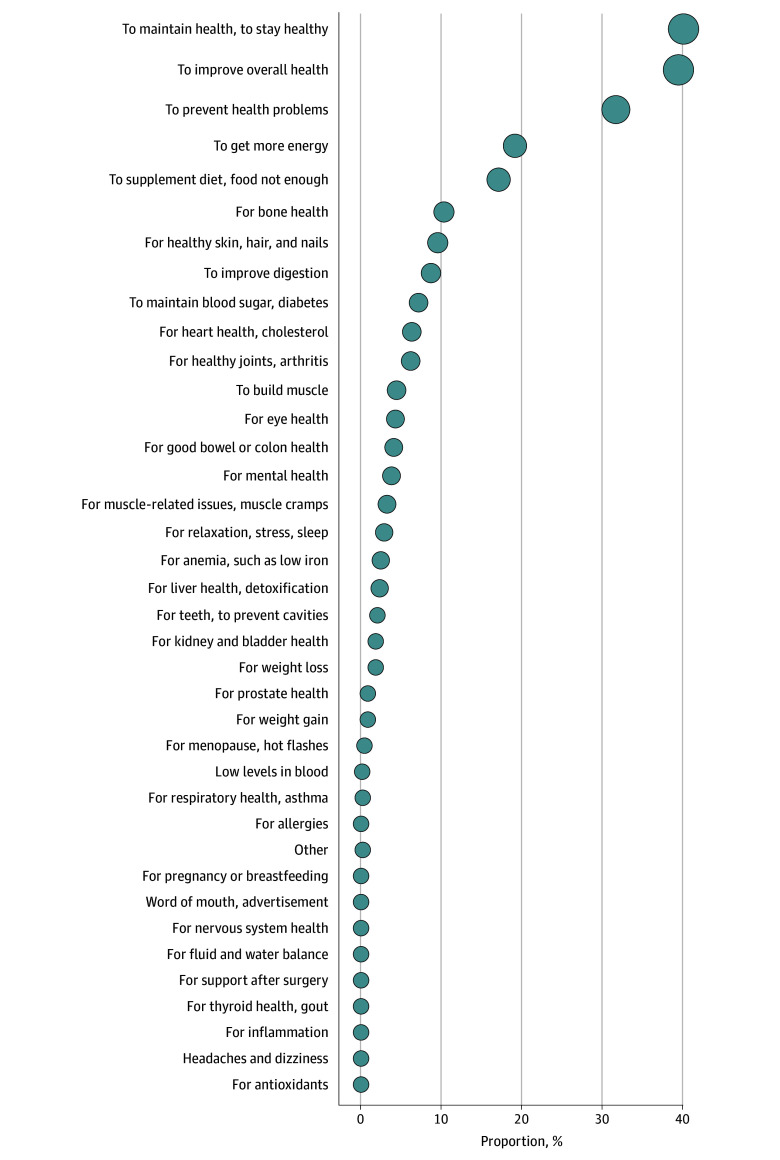
Additional Reasons for Dietary Supplement Use Among Those Using Supplements for Perceived Immune Benefits Among those taking a dietary supplement for perceived immune benefits (1487 individuals), the percentages of persons who reported using the dietary supplement for each additional reason are shown here. The most common reason for dietary supplement usage in addition to perceived immune benefits was “to maintain health, to stay healthy.” The size of each circle corresponds to the percentage of persons.

Among those taking a dietary supplement for perceived immune benefits, 60.1% (95% CI, 54.0%-65.9%) took a dietary supplement whose label had claims related to immune benefits. No differences by sex, age, race and Hispanic origin, income, education, food security, self-rated diet quality, or self-rated overall health were observed. Only 16.6% (95% CI, 13.7%-19.8%) took a dietary supplement for perceived immune benefits based on a health care professional’s recommendation, but doing so increased with age and declining self-rated health (Table).

## Discussion

This study has 3 findings in a prepandemic context. First, approximately 1 in 9 US residents used a dietary supplement for perceived immune benefits, and such usage varied by several sociodemographic and health characteristics. Second, label claims related to immune benefits consistently appeared on over half of dietary supplements taken for perceived immune benefits. Lastly, the prevalence of dietary supplement use for perceived immune benefits due to a doctor recommendation and dietary supplement use exclusively for perceived immune benefits were both generally low, but both increased among older adults and individuals in poorer health. Patterns by sex, race, and Hispanic origin differed between those taking a dietary supplement for perceived immune benefits compared with previous estimates for overall dietary supplement use.^1^

Limitations include potential recall bias and measurement error due to reliance on self-reported data and potentially misidentified products. Strengths include using nationally representative data and detailed dietary supplement collection in NHANES. In conclusion, because many US residents use dietary supplements for perceived immune benefits, continued research in diverse populations is needed to determine their clinical relevance. Additionally, while dietary supplements can help improve nutrient intake, upstream nutritional disparities must also be addressed.

## Notes

Supplement 1.
eMethods.

Supplement 2.
Data Sharing Statement
