# Supplementary material for: Prepandemic Prevalence of Dietary Supplement Use for Immune Benefits
Source: JAMA Netw Open. 2025 Feb 11;8(2):e2459291. doi: 10.1001/jamanetworkopen.2024.59291 (PMC11815518; doi:10.1001/jamanetworkopen.2024.59291)
Supplement: Supplement 1. — eMethods. [file jamanetwopen-e2459291-s001.pdf]

Supplemental Online Content

Nagai-Singer MA, Wambogo EA, Pasiakos SM, Gahche JJ. Prepandemic prevalence of dietary supplement use for immune benefits. *JAMA Netw Open.* 2025;8(2):e2459291. doi:10.1001/jamanetworkopen.2024.59291

eMethods

This supplemental material has been provided by the authors to give readers additional information about their work.

© 2025 Nagai-Singer MA et al. *JAMA Network Open*.

Ethical Review of Study and Informed Consent

The National Center for Health Statistics (NCHS) Ethics Review Board approved NHANES. Adults 18+ years provided consent, and adolescents 12-17 years provided documented assent. All participants aged ≥16 years or emancipated minors self-reported, and a proxy answered for those <16 or those who were not able to answer themselves. Parental permission was also obtained for adolescents and younger children. All data used in the current study are publicly available and were obtained from NHANES, which follows the appropriate ethical and informed consent practices.

NHANES Data

NHANES is nationally representative, providing a comprehensive overview of the population's DS use. DS data in NHANES are collected using multiple methods, allowing for collection of information on all DS products used, including episodically used DSs. NHANES only includes DSs as defined by the Dietary Supplement and Education Act of 1994, which excludes homemade formulations. Details of the NHANES study design, implementation, data sets, analytic considerations, and other documentation are available online.[1]

The data used to assess DS use were collected during the home interview using the Dietary Supplements and Prescription Medication Questionnaire (DSQ). The interview response rate was 51%.[2] Participants were asked to show all containers for DSs used in the past 30 days. Product labels for all DSs reported were then obtained by a nutritionist post-interview. During data processing, the NCHS assigns match codes depending on the level of confidence that an exact product was found in their DS database. To be eligible for this analysis, the DS must have a matching code of 1 (Exact or Near Match), 2 (Probable Match), or 4 (Reasonable Match). DSs

© 2025 Nagai-Singer MA et al. *JAMA Network Open*.

with a matching code of 3 (Generic Match), 5 (Default Match), or 6 (No Match) were excluded from this analysis. The DSQ includes a question on the reason(s) why the participant is taking each DS reported. This variable was used to identify DSs that were used “to prevent colds, boost immune system” (perceived immune benefits). DSs used for this purpose were mostly vitamin and/or mineral DSs without botanical ingredients. To understand if perceived immune benefits was the main motivation for using these DSs, the study analyzed if perceived immune benefits was the sole reason reported by participants. Participants also reported if they used a DS due to the recommendation of a doctor or other healthcare provider.

Dietary Supplement Label Database (DSLD) Search

The DSLD was used to identify label claims related to immune benefits on the eligible DSs reported in NHANES to be used for perceived immune benefits. To match the DSs reported in NHANES to the correct label in the DSLD, we matched the ingredient information provided in the NHANES Dietary Supplement Database – Ingredient Information (DSII) to the ingredient information found on the labels cataloged in the DSLD.

First, in the DSLD, the brand name of the supplement (e.g., Equate) was applied as a filter, and the supplement name (e.g., Children’s Multivitamin Gummies) was entered in the search bar. If the DSLD did not recognize the brand name as a filter, then the brand name and the supplement name were entered into the search bar together (e.g., Equate Children’s Multivitamin Gummies). Search results were sorted by best match. The first result in which the ingredient information on the DSLD label matched the DSII information was selected as the label for that supplement, but if the search yielded multiple accurate matches, all were examined for label claims related to immune benefits. If labeling discrepancies between matches were found, we

© 2025 Nagai-Singer MA et al. *JAMA Network Open*.

defaulted to using the label with the immune claims. If an eligible supplement reported in NHANES was not available in the DSLD, label claims related to immune benefits were determined through the name of the supplement. For example, a DS named "Immune Plus System Support" that could not be matched to a label in the DSLD would be considered to have a label claim related to immune benefits. DSLD searches were conducted from 11/20/2023 to 01/04/2024.

Sociodemographic Variables

Age was categorized into four groups: 0-11, 12-19, 20-59, and 60+ years. Race and Hispanic origin were self-reported by the participant according to the classifications provided by the NHANES survey; "other" included persons who reported multiple races. Race and Hispanic origin were assessed in this study to investigate differences in prevalence based on these characteristics. Family income was defined based on the Federal Poverty Level (FPL) and was categorized as FPL <130%, 130%-350%, and >350%. The recommended threshold for eligibility for the Supplemental Nutrition Assistance Program and the free and reduced-price school lunch program is 130%.[3,4] Educational attainment for adults was split into two groups (high school graduate/equivalent GED or less, and some college or college graduate). Household food security categories were based on responses to the U.S. Food Security Survey Model questions, and low and very low were combined into one group. Self-rated diet quality and self-rated overall health for participants aged 16+ years were split into three groups (excellent/very good, good, and fair/poor).

Statistical Analyses

© 2025 Nagai-Singer MA et al. *JAMA Network Open*.

All statistical analyses were performed with SAS (version 9.4, SAS Institute Inc, Cary, NC)[5] and SUDAAN (version 11.0, RTI International, Research Triangle Park, NC).[6] SAS survey procedures were used to account for the NHANES complex survey design, using procedures that do not assume a simple random sample. The interview weights were used for all analyses to account for oversampling and survey nonresponse and are post-stratified to U.S. Census Bureau population estimates. Weights account for non-response at both the respondent and item level, plus non-coverage, and day of the week data collection. Prevalence estimates are provided for types of DS and motivation(s) for use. Standard errors were calculated using Taylor linearization and 95% Confidence Intervals were estimated using the Clopper-Pearson CI adopted for complex surveys by Korn and Graubard.[7] Prevalence estimates were obtained for each variable independently using SUDAAN Proc Descript. Statistical testing of differences within unordered covariates were tested using t-tests. For ordered covariates, tests for linear trends were examined using orthogonal polynomials, with statistical significance set at $P < 0.05$. All estimates of proportions were evaluated using the NCHS data presentation standards for proportions, and values presented have a relative SE < 30% unless otherwise noted.[7] For all variables, the rate of missing data was less than 10%, and estimates for those with missing data are not shown. All analyses were performed from January to July of 2024.

STROBE Guidelines

This report follows the STROBE reporting guidelines for cross-sectional studies.

References

1. Statistics CNCfH. 2017-March 2020 Pre-Pandemic Dietary Data - Continuous NHANES. Accessed 02/27/2024, https://wwwn.cdc.gov/nchs/nhanes/search/datapage.aspx?Component=Dietary&Cycle=2017-2020

© 2025 Nagai-Singer MA et al. *JAMA Network Open*.

2. Statistics CNCfH. NHANES Response Rates and Population Totals. Accessed 02/27/2024, https://wwwn.cdc.gov/nchs/nhanes/ResponseRates.aspx
3. U.S. Department of Agriculture FaNS. SNAP Eligibility. Accessed 03/12/1997, https://www.fns.usda.gov/snap/recipient/eligibility
4. U.S. Department of Agriculture FaNS. Child Nutrition Programs: Income Eligibility Guidelines (2023-2024). Accessed 03/12/2024, https://www.fns.usda.gov/cn/fr-020923#:~:text=The%20Income%20Eligibility%20Guidelines,-The%20following%20are&text=The%20Department's%20guidelines%20for%20free,to%20the%20next%20whole%20dollar
5. *SAS [computer program]. Version 9.4.* 2013.
6. *SUDAAN. Release 11.0.* . 2012.
7. Parker JD, Talih M, Malec DJ, et al. National Center for Health Statistics Data Presentation Standards for Proportions. *Vital Health Stat 2*. Aug 2017;(175):1-22.

© 2025 Nagai-Singer MA et al. *JAMA Network Open*.
