# Supplementary material for: Prepandemic Prevalence of Dietary Supplement Use for Immune Benefits
Source: JAMA Netw Open. 2025 Feb 11;8(2):e2459291. doi: 10.1001/jamanetworkopen.2024.59291 (PMC11815518; doi:10.1001/jamanetworkopen.2024.59291)
Supplement: Supplement 2. — Data Sharing Statement [file jamanetwopen-e2459291-s002.pdf]

Data Sharing Statement

Nagai-Singer. Prepandemic Prevalence of Dietary Supplement Use for Immune Benefits. *JAMA Netw Open*. Published February 11, 2025. doi:10.1001/jamanetworkopen.2024.59291

Data
Data available: Yes
Data types: Deidentified participant data, Other (please specify)
Additional Information: Other: merged dataset
How to access data: Deidentified participant data from the National Health and Nutrition Examination Survey (NHANES) is available at https://wwwn.cdc.gov/nchs/nhanes/continuousnhanes/default.aspx?Cycle=2017-2020, and data from the Dietary Supplement Label Database is available at https://dsld.od.nih.gov/.
When available: With publication

Supporting Documents
Document types: Statistical/analytic code
How to access documents: Merged dataset and statistical code will be made available upon request to jaime.gahche@nih.gov
When available: With publication

Additional Information
Who can access the data: Anyone requesting the data
Types of analyses: For any purpose
Mechanisms of data availability: Any mechanism
Any additional restrictions: No restrictions, merged dataset includes all publicly available datasets.
